# Aging, Melatonin, and the Pro- and Anti-Inflammatory Networks

**DOI:** 10.3390/ijms20051223

**Published:** 2019-03-11

**Authors:** Rüdiger Hardeland

**Affiliations:** Johann Friedrich Blumenbach Institute of Zoology and Anthropology, University of Göttingen, 37073 Göttingen, Germany; rhardel@gwdg.de; Tel.: +49-551-395414

**Keywords:** circadian, immunosenescence, inflammaging, melatonin, microRNAs, sirtuin-1

## Abstract

Aging and various age-related diseases are associated with reductions in melatonin secretion, proinflammatory changes in the immune system, a deteriorating circadian system, and reductions in sirtuin-1 (SIRT1) activity. In non-tumor cells, several effects of melatonin are abolished by inhibiting SIRT1, indicating mediation by SIRT1. Melatonin is, in addition to its circadian and antioxidant roles, an immune stimulatory agent. However, it can act as either a pro- or anti-inflammatory regulator in a context-dependent way. Melatonin can stimulate the release of proinflammatory cytokines and other mediators, but also, under different conditions, it can suppress inflammation-promoting processes such as NO release, activation of cyclooxygenase-2, inflammasome NLRP3, gasdermin D, toll-like receptor-4 and mTOR signaling, and cytokine release by SASP (senescence-associated secretory phenotype), and amyloid-β toxicity. It also activates processes in an anti-inflammatory network, in which SIRT1 activation, upregulation of Nrf2 and downregulation of NF-κB, and release of the anti-inflammatory cytokines IL-4 and IL-10 are involved. A perhaps crucial action may be the promotion of macrophage or microglia polarization in favor of the anti-inflammatory phenotype M2. In addition, many factors of the pro- and anti-inflammatory networks are subject to regulation by microRNAs that either target mRNAs of the respective factors or upregulate them by targeting mRNAs of their inhibitor proteins.

## 1. Introduction

Aging is associated with manifold changes. These comprise declined secretion of hormones such as melatonin [1,2], reduced activities of aging-related factors such as sirtuin-1 (SIRT1) [3], deterioration of the circadian oscillator system [4,5], multiple alterations in the immune system that is frequently shifted toward the proinflammatory side [6,7,8,9,10], and many more deviations of cell biological relevance. Importantly, the changes specifically mentioned are interrelated in multiple ways [3,5,9,10]. This is largely based on the pleiotropy of both melatonin [11] and the circadian system [12,13,14]. However, these relationships are highly complex, include actions in opposite directions, and cannot be interpreted in reductionist ways.

For example, the effects of melatonin in the immune system can be either pro- or anti-inflammatory [15,16,17]. Generally, melatonin acts as an immune stimulatory agent and the direction into which the balance is shifted has turned out to be highly conditional. The influence of melatonin on SIRT1 expression has also revealed effects of either down- or upregulation, in this case, with a remarkable difference between tumor and non-tumor cells [5]. While being strongly suppressive in cancer, melatonin mainly stimulated SIRT1 in nontransformed cells, especially in the context of aging. However, a considerable problem exists concerning the, unfortunately, prevailing determination of SIRT1 expression rather than activity. Assuming a correlation between expression and activity, which is questionable, is a profound misconception in the case of sirtuins [18]. Sirtuin activities are not primarily determined by their protein levels but rather by NAD^+^ concentration, which depends on the activity of nicotinamide phosphoribosyltransferase (NAMPT) [19,20,21,22]. The contrast between SIRT1 expression and activity has become evident in a study on the effect of BRCA1 (breast cancer 1, early onset) in ovarian cancer [23]. Suppression of BRCA1 reduced SIRT1 expression, but it increased NAD^+^ concentration and, therefore, SIRT1 activity. Moreover, BRCA1 overexpression upregulated SIRT1 expression and decreased NAD^+^ levels and SIRT1 activity. This divergence also seems to be relevant to aging. SIRT1 expression was not generally shown to be decreased in the course of aging, but rather, it was shown to often increase. Nevertheless, SIRT1 activity was found to be reduced because of lowered NAD^+^ levels [24,25]. However, this does not yet mean that positive correlations between SIRT1 expression and activity are generally excluded in the context of aging. In senescence-accelerated SAMP8 mice, SIRT1 expression was found to be reduced relative to the widely isogenic control strain SAMR1 [26]. Moreover, a number of studies have shown that effects of melatonin that increased SIRT1 expression were suppressed by sirtuin inhibitors such as sirtinol or EX527 [27,28,29,30,31,32,33,34,35,36,37,38,39]. Therefore, mandatory requirements are to either determine SIRT1 activity and NAD^+^ concentration (recommended) or to at least test the effects of sirtuin inhibitors [18].

The changes in melatonin secretion and SIRT1 activity also have a circadian dimension. Both of them are under circadian control and exhibit cycles of high amplitudes. Moreover, either of them can influence circadian oscillators. Apart from its known chronobiotic actions via the suprachiasmatic nucleus (SCN), melatonin also influences peripheral oscillators [40]. SIRT1 has been identified as an accessory oscillator component [19,20,21] that increases circadian amplitudes of both central and peripheral clocks [21,41]. At least one of the mechanisms described is of relevance for antagonizing age-related decreases in the amplitudes of the SCN output [41]. An additional aspect concerns the conclusion that SIRT1 acts as a partial mediator of melatonin effects [18,42]. Finally, it seems important to remain aware of the different phases of increases and decreases observed within a circadian cycle. It is basic knowledge of chronobiology that a specific treatment applied in different phases leads to different, often opposite, effects [5]. In the context of aging, additional difficulty results from the fact of non-identical changes observed between the populations of oscillators within the body. In a senescent mammal, some oscillators exhibit phase changes, others reduced amplitudes, in the extreme, down to arrhythmicity, whereas others remain widely unchanged [4]. Collectively, all these variabilities summarized here oppose any expectation of finding exclusively unidirectional relationships by applying melatonin, sirtuin overexpression, or other modulators of circadian rhythms.

These reservations also have to be kept in mind when considering the effects of melatonin and SIRT1 in the regulation of pro- and anti-inflammatory processes. These are of particular relevance to aging and age-related diseases, especially as proinflammatory mechanisms gain increasing importance in the course of senescence. The overlapping effects of melatonin and SIRT1 in the field of inflammation regulation will be discussed in this article.

## 2. Melatonin and the Proinflammatory Network

In the course of studies that revealed numerous effects of melatonin beyond the control of circadian oscillators, immunological actions of melatonin were also discovered [9,15,43,44,45,46]. However, the statement that melatonin possesses properties that exceed the control of oscillators does not mean that the resulting effects are independent of circadian rhythms. Both melatonin secretion and signaling are subjected to circadian control. Parameters affected, including immunological ones, are also influenced by the circadian system [11,47]. With regard to the immune system, the situation is somewhat more complicated as melatonin is also synthesized by several types of leukocytes [11,44,48,49].

Many of the earlier investigations on melatonin in the immune system revealed proinflammatory effects, as repeatedly summarized [11,15,17,44]. Notably, these actions were predominantly discovered in studies using isolated leukocytes or transformed leukocyte-derived cell lines [16,17,44]. Although such an approach appears to be reasonable in the beginning, it may not sufficiently reflect the complexity of the immune system and the additional participation of, in classical terms, non-immune cells in both proinflammatory responses and the regulation of the pro-/anti-inflammatory balance. Data obtained in humans also indicated proinflammatory actions of melatonin in rheumatoid arthritis [50,51,52]. These findings were regarded as a general caveat concerning the use of the immune stimulator melatonin in autoimmune diseases. This view has received partial support by a study in a multiple sclerosis model [53]. Most of the earlier findings on proinflammatory effects in cell cultures concerned the upregulation of proinflammatory cytokines (IL-1β, IL-2, IL-6, IL-8, IL-12, IFNγ, and TNFα) and downregulation of their anti-inflammatory counterpart (IL-10), as summarized elsewhere [15,16,44]. Additionally, melatonin was shown to counteract the inhibition of IL-2 production by prostaglandin E_2_ in human lymphocytes [54].

However, this short overview of the inflammation-promoting effects does not yet sufficiently describe the actions of melatonin within the proinflammatory network. In fact, these actions also comprise numerous suppressive changes in this network and, thereby, can turn out to result in an anti-inflammatory balance. To appropriately judge the complex influences of melatonin, it is necessary to view it under these different, additional perspectives: (1) changes in tissues and their main cellular constituents; (2) cell specificity; (3) effects on clonal expansion, differentiation, and polarization in the immune system; (4) contributing proinflammatory effects of non-immune cells; and (5) conditionality, with regard to high-grade or low-grade inflammatory challenges that may occur in the progression of aging or by experimental procedures.

As recently summarized, melatonin-induced elevation of proinflammatory cytokines, such as IL-1β, IL-2, IL-6, IL-12, TNFα, and IFNγ, has been repeatedly observed in monocytes, monocyte-derived cell lines, and type 1 T-helper cells [17]. One can assume that similar responses occur in M1 macrophages, M1 microglia and, especially concerning IL-6, IL-8, and TNFα, also in epithelial cells and various other cell types. Notably, these findings can be seen in the context of pro-oxidant and cytotoxic effects exerted by melatonin in monocytes, as observed above an activation threshold as low as 50 pM [55]. Moreover, melatonin was found to increase another proinflammatory cytokine, IL-17A, in Th17 cells [56], an effect that spreads proinflammatory responses by inducing the release of other mediators, such as IL-1β, IL-6, TNFα, the neutrophil-attracting Il-8, and by upregulating cyclooxygenase-2 (COX-2) and iNOS (inducible NO synthase) [57,58,59,60,61,62,63,64,65]. This host of secondary effects by IL-17A has been shown to be involved in autoimmune diseases and in neuroinflammation and is, therefore, of relevance to age-related health problems and to adverse effects of melatonin, especially in autoimmunity. Additionally, melatonin was shown to upregulate IL-1β, TNFα, and IFNγ in splenocytes [66]. Several other effects of melatonin concern the upregulation of cytokines that are primarily involved in differentiation and clonal expansion, such as M-CSF (macrophage colony-stimulating factor) and SCF (stem cell factor) in macrophages and splenocytes, TGFβ (transforming growth factor) in macrophages and dendritic cells, and thymosin-α and thymulin in thymocytes, as summarized elsewhere [16]. Melatonin was shown to promote differentiation of progenitor cells to Th lymphocytes, NK lymphocytes, granulocytes, and macrophages [16,44,45]. All these effects may have secondary consequences to the pro-/anti-inflammatory balance. In bone marrow, an upregulation of GM-CSF (granulocyte/monocyte colony-stimulating factor) may be interpreted in a similar way, but, on the other hand, melatonin-induced release of the immune-opioids MIO15 (melatonin-induced opioid) and MIO67 [16,67,68,69] can be expected to favor the anti-inflammatory side of the immune system.

The proinflammatory network comprises numerous components, which are regulated differently by melatonin. Moreover, the responses are often highly contextual. Findings obtained by applying pro-oxidant or proinflammatory challenges, such as administration of oxidotoxins, mitotoxins, bacterial lipopolysaccharides (LPS) or experimental sepsis, may not lead to results comparable to those found in aging. Therefore, it seems important to focus on changes that really occur in aging or age-related diseases and can be mostly classified as low-grade inflammation [10,17,70]. Several sources of aging-related inflammation shall be mentioned as being of foremost importance: (1) enhanced release of proinflammatory cytokines as a consequence of immunosenescence; (2) neuronal overexcitation in an ^•^NO-mediated interplay with microglia and astrocytes; (3) amyloid-β (Aβ) toxicity; (4) brain insulin resistance; (5) diabetes and metabolic syndrome; (6) senescence-associated secretory phenotype (SASP) of non-immune cells with DNA damage response (DDR); (7) garb-aging; (8) reduced antioxidant protection by decreased melatonin; and, presumably, also (9) metabolic malfunction because of poorly coordinated and weakened circadian rhythms. Notably, all these pathophysiological alterations are associated with oxidative and nitrosative/nitrative stress and mitochondrial malfunction. They have multiple interconnections and, thereby, display the potential of forming vicious cycles [10,17,70]. 

Immunosenescence, which is primarily associated with thymic involution and, additionally, exhaustion of leukocyte subpopulations upon lifelong exposure to foreign antigens, typically leads to a shift toward a proinflammatory phenotype, which is evident by increased levels of proinflammatory cytokines that may result in a so-called immune risk profile [7,10,70,71,72,73]. Neuronal overexcitation causes increased formation of ^•^NO, which can activate microglia, astrocytes, and other neurons that also release ^•^NO at elevated rates. These responses contribute to a spreading of excitation and to the release of oxidants and proinflammatory mediators, especially by microglia. High levels of ^•^NO and superoxide anions (O_2_^•−^) generate the formation of their adduct, peroxynitrite (ONOO^−^), a highly reactive intermediate that causes damage to mitochondria and additionally generates free radicals (ONOO^−^ + H^+^ → ONOOH → ^•^NO_2_ + ^•^OH; ONOO^−^ + CO_2_ → ONOOCO_2_^−^ → ^•^NO_2_ + CO_3_^•−^) [74,75]. These radicals, their precursors, and several other reactive nitrogen species that can be formed from them interfere with the mitochondrial electron transport chain (ETC) [74,76,77]. As recently summarized, melatonin protects the ETC against these reactive intermediates [77], in addition to various other mitochondria-protecting actions [78,79,80,81,82,83]. Aβ peptides have, besides other effects, pro-oxidant and proinflammatory properties. The main toxicity is caused by Aβ monomers and oligomers, with an additional contribution by amyloid plaques. Peptides and oligomers induce microglia activation [84,85] and, additionally, responses by astrocytes [86] and neurons [86,87], which all upregulate NADPH oxidase, thereby elevating O_2_^•−^ formation. Notably, the release of proinflammatory mediators in response to Aβ is not restricted to microglia. Even neurons have been shown to respond to Aβ peptides by upregulating TNFα, IL-1β, COX-2, and the T-cell and monocyte attractant chemokine CX3CL1 [87]. Aβ toxicity should not only be seen in the context of established Alzheimer’s disease (AD), since these peptides also appear in the CSF (cerebrospinal fluid) of healthy subjects, but are normally widely removed by clearance of CSF and ISF (interstitial fluid), especially during sleep [88,89,90,91]. Sleep disturbance impairs Aβ clearance, which has been discussed as a contribution to the development of AD pathology [92]. Circadian disruption, a major source of sleep disturbances, indicates that changes in melatonin secretion may be involved. In fact, melatonin has been recently shown to increase Aβ clearance [93,94]. Numerous other anti-amyloidogenic effects of melatonin have been also described and repeatedly reviewed [70,95,96,97,98]. A recent overview [17] has particularly focused on the anti-inflammatory aspect and the suppression of Aβ secretion by melatonin. This includes, in cellular test systems, the reduction of βAPP (β-amyloid precursor protein) mRNA expression [99], inhibition of β- and γ-secretases [100], and the upregulation of α-secretase, an enzyme that competes with β- and γ-secretases and produces the nonamyloidogenic and neuroprotective fragment sAPPα [101]. The shift from β- and γ-secretases to α-secretase was recently also confirmed in the hippocampus of senescent mice [102,103]. In transgenic AD mouse models, melatonin was found to substantially delay the accumulation of Aβ and to extend lifespan [104,105]. However, this was only observed after an early onset of treatment in the first months of life, but not at later age [106], findings that leave a pessimistic perspective for the treatment of humans. Another, surprising nexus to AD pathology and inflammation emerged, when brain insulin resistance was shown to be an early sign of neuroinflammation at the onset of AD [107,108]. For further details and references, see [17,109]. This aspect remains to be studied in the context of melatonin, but would require a strict discrimination between nocturnal rodents and humans, since the disregard of this difference has led to substantial misinterpretations concerning the beneficial or detrimental role of melatonin in type 2 diabetes [5,16]. Also, beyond the CNS, type 2 diabetes and, in a broader sense, metabolic syndrome, are associated with low-grade inflammation, a phenomenon known under the term of metaflammation [110,111,112], which contributes to inflammaging [111,112]. Numerous beneficial effects of melatonin in metabolic syndrome have been recently summarized [113], which have been observed in both experimental animals and clinical studies and indicate a reduction of metaflammation.

Another aspect of proinflammatory effects concerns the role of non-immune cells in inflammaging. This comprises mainly three processes, SASP, garb-aging, and release of macromolecules from dying cells. In SASP, DNA-damaged cells are arrested in terms of proliferation, but continue to participate in the metabolism of the tissue. However, they release several signal molecules, including proinflammatory cytokines and chemokines, thereby inducing local, low-grade inflammation [114,115,116,117,118]. SASP has been observed in many cells of peripheral tissues, but also in astrocytes and is, therefore, relevant to the CNS [70,119,120]. Notably, SASP has also been shown to be induced by sleep disruption [121]. As this was associated with increased oxidative stress, the observed changes may comprise a chronodisruption-related nocturnal loss of protection by melatonin. Studies on direct effects of melatonin against SASP are still in their infancy, but recent initial results have described this [122,123,124]. In mechanistic terms, melatonin was shown to suppress the PARP-1 [poly(ADP-ribose) polymerase-1]-induced expression of SASP genes [122]. Whether or not this role of PARP-1 can be generalized remains to be clarified, since this regulator, which is an indicator of DNA damage, has also been interpreted as an epigenomic safeguard that interferes with SASP-associated microRNAs [125]. In another context, melatonin was reported to suppress SASP by downregulating NF-κB and upregulating Nrf2 (nuclear factor erythroid 2-related factor 2) [124], i.e., two known effects of melatonin that are typically observed in mechanisms of antioxidative and anti-inflammatory protection [17,126]. Other sources of low-grade inflammation by non-immune cells, such as garb-aging [127] and release of components of dying cells such as histone H1 [128] and molecules of mitochondrial origin (nucleic acids, *N*-formylated peptides and proteins) to the cytosol [129,130,131,132], are presumably also of substantial relevance to inflammaging, but have not been sufficiently studied in relation to melatonin. Nevertheless, this connection exists with high likelihood with regard to mitochondrial DNA (mtDNA), especially if it is damaged or oxidized. Activation of the NLRP3 inflammasome by mtDNA has been repeatedly described, whereas counteractions by melatonin have been multiply documented and reviewed, as summarized elsewhere [133]. Melatonin administration was shown to be as effective as NLRP3 deficiency [134]. Unfortunately, the important proinflammatory pathway of cGAS/STING (cyclic GMP-AMP synthase/stimulator of IFN genes) signaling has, to date, been poorly considered in melatonin research. This would be of particular interest, as cGAS is a cytosolic DNA detector. Moreover, the depletion of cGAS and STING counteracted cell senescence and prevented SASP in human and murine fibroblasts [135]. cGAS appears as a mediator of cell stress reactions and has been assumed to be involved in aging-related diseases [131]. This interpretation has received support by recent findings on a human *Sting* gene polymorphism that is associated with low-risk of aging-related diseases, presumably by reducing inflammaging [136].

Activation of the NLRP3 inflammasome in various systems, under different conditions and counteractions by melatonin, have been recently reviewed [17]. These findings were widely related to the suppression of NF-κB signaling by melatonin, which is likewise important in the attenuation of oxidative damage [126]. NF-κB was also reported to induce pyroptosis via gasdermin D (GSDMD) in adipose tissue, which was likewise inhibited by melatonin [137]. Other inflammation-related and melatonin-sensitive effects of NF-κB concern the upregulation of iNOS and COX-2 [138,139,140,141]. Moreover, in the context of presenilin-1 upregulation and pathogenic βAPP processing, a pathway involving PIN1 (peptidyl-prolyl cis-trans isomerase NIMA-interacting 1) and GSK3β (glycogen synthase kinase 3β) was shown to activate NF-κB, which was, in accordance with many other findings on NF-κB suppression, inhibited by melatonin [142]. 

Another proinflammatory route is based on TLR4 (toll-like receptor 4) activation, e.g., via the IFNγ adaptor protein, TRIF (toll-receptor-associated activator of interferon). In the macrophage-like cell line RAW264.7, melatonin has been shown to suppress the release of proinflammatory cytokines, such as TNFα, IL-1β, IL-6, and IL-8, by TRIF and TLR4 inhibition [143]. As TLR4 also mediates pro-oxidant actions via NF-κB, more general effects by melatonin on this pathway may be assumed. This conclusion is supported by several pertinent findings describing protection by melatonin [17]. Similar anti-inflammatory effects were also obtained in an in vivo model of ovarian cancer [144]. Information on melatonin effects concerning other TLR subforms is still scarce. No effects were found in a single study on TLR2 [144], whereas inhibition of TLR3 was reported [145,146].

A further possible proinflammatory pathway that is inhibited by melatonin concerns mTOR (mechanistic target of rapamycin) activation. However, most respective information is not directly related to inflammation, but rather to mitophagy or apoptosis. Interestingly, an mTOR inhibiting action by melatonin was also shown to be suppressed by inhibition of PIN1 [123]. Moreover, the attenuation of microglial activation and neuroinflammation after traumatic brain injury by melatonin was also interpreted on the basis of interference with mTOR [147]. This route will be of further interest in the specific context of melatonin’s anti-inflammatory actions.

## 3. Melatonin, SIRT1, and the Anti-Inflammatory Network

While melatonin is partially acting by either stimulating or inhibiting components of the proinflammatory network, it also upregulates molecules of an anti-inflammatory network. Some of them are negatively correlated with proinflammatory agents. For instance, NF-κB, a transcription factor involved in prooxidant and, thereby, proinflammatory responses, is inversely coupled to antioxidant and anti-inflammatory regulators, in particular, Nrf2 [17,126,139,148,149,150,151]. A similar correlation seems to exist in the case of PARK7 (parkinsonism associated deglycase; also known as DJ-1) [149,150], a protein that acts, beside other effects, as a redox-sensitive chaperone and stress sensor. In Parkinson’s disease (PD), it has been shown to be neuroprotective [152]. 

An especially important anti-inflammatory regulator under control by melatonin is SIRT1. It has been classified as a secondary signaling molecule that mediates several effects of melatonin [18,42]. In non-tumor cells, it has been shown to be upregulated by melatonin and effects by melatonin have been repeatedly reported to be suppressed by sirtuin inhibitors or *Sirt1* siRNA [5], notably also in an anti-inflammatory context [17]. The relationship between melatonin and SIRT1 may be regarded as a mutual one, since SIRT1 can enhance circadian amplitudes in the SCN [41] and may, thereby, influence the melatonin rhythm [3]. With this background, the functional overlap of described melatonin and SIRT1 actions seems worthwhile to be recalled. 

This overlap becomes obvious from two lines of evidence, (1) the interference of sirtuin-related agents with melatonin effects, and (2) similar actions of melatonin and SIRT1. In the former context, reductions of NLRP3 inflammasome activation and IL-1β levels by melatonin were blocked by the sirtuin inhibitor EX527 in a rat COPD (chronic obstructive pulmonary disease) model [39]. The same inhibitor also blocked anti-inflammatory actions of melatonin such as downregulation of TNFα and IL-1β in acute kidney injury of rats [36]. Several other results on sirtuin inhibition of melatonin treatment were obtained under conditions of more severe inflammation. This was observed in cardiac ischemia/reperfusion of normal [30] and diabetic rats [31], correspondingly in ER stress of H9C2 cardiomyocytes [31], in LPS-treated microglial cell lines [37], and in brain injury by cecal ligation/puncture in mice [32]. Further studies, without measurement of inflammatory parameters, in which melatonin effects were blunted by sirtuin inhibitors or *Sirt1* siRNA, are summarized elsewhere [5,17].

Numerous studies have demonstrated antioxidant and anti-inflammatory actions by SIRT1 that are also known from melatonin [17]. This concerns the suppression of NF-κB activation [153,154,155,156], the upregulation of Nrf2 [157,158,159,160,161], suppression of NLRP3 inflammasome activation [39,162,163,164,165], and inhibition of TLR4 signaling [166,167,168]. An important player in TLR4 activation is HMGB1 (high mobility group box-1), an inflammatory signaling molecule released by monocytes and macrophages, and also by other cells (e.g., endothelial). SIRT1 is known to deacetylate HMGB1 [169,170], to inhibit its nucleocytoplasmic transfer, and to prevent its release [167,171,172,173,174,175,176]. Importantly, HMGB1 also favors the polarization of macrophages and microglia towards the proinflammatory M1 type [177,178,179,180,181]. With regard to the melatonin–SIRT1 relationship, it is of interest that anti-inflammatory actions via HMGB1 inhibition have been also reported for melatonin, as recently summarized [17]. Again, SIRT1 may mediate melatonin effects in this case, an assumption to be experimentally confirmed.

SIRT1 also displays several additional anti-inflammatory effects, which cannot yet be matched with corresponding data from melatonin treatment. As mentioned in the previous section, some reports on melatonin effects on mTOR signaling have not been directly related to inflammation. Other studies on actions of melatonin on mTORC1 (mTOR complex 1) activity in cancer cells led to contradictory results [182,183,184] and, therefore, do not provide a reliable basis for comparison. However, SIRT1 has been shown to counteract adipose inflammation by suppressing mTORC1 signaling [185]. Another investigation on liver steatosis reported an increase of mTORC1 activity upon hepatocyte-specific deletion of SIRT1 [186]. Under conditions of sepsis, SIRT1 was also reported to deacetylate and, thereby, inhibit NICD (intracellular domain of Notch) [187,188]. Apart from its developmental roles, Notch is known to act in a proinflammatory way by promoting M1 polarization of macrophages or related cells. Conversely, SIRT1 knockdown upregulated Notch1 in stellate cells at both mRNA and protein levels [189]. However, the relationship to melatonin remains unclear because beneficial effects of Notch signaling were reported in studies on protection against ischemia/reperfusion and Aβ toxicity by melatonin [190,191,192], in spite of the fact that Notch activation is known to cause inflammatory responses. Either the endpoints studied were unrelated to macrophages or microglia, respectively, or Notch signaling may be a case in which SIRT1 does not mediate melatonin effects. This would be surprising, as melatonin was shown to upregulate SIRT1 under conditions of ischemia/reperfusion, and as some of the melatonin effects were blocked by EX527 or *Sirt1* siRNA [5,30,31].

Another recently reported anti-inflammatory action of SIRT1 has been observed in macrophages after treatment with LPS. The lncRNA-CCL2, which is related to the gene locus of the chemokine CCL2 and stimulates the release of proinflammatory cytokines, was downregulated by SIRT1 [193]. No corresponding data on changes of lncRNA-CCL2 expression by melatonin are actually available. 

Despite the fact that many proinflammatory effects of melatonin are mediated by lymphocytes [15,44,49,54,56], such responses were also obtained in monocytes and monocyte-derived cells [11,15,44]. However, as macrophages and related cells represent major executive players in inflammation, their polarization into proinflammatory M1 or anti-inflammatory M2 phenotypes is of utmost importance for the pro-/anti-inflammatory balance. In fact, melatonin is capable of shifting this balance toward the anti-inflammatory side by favoring M2 and disfavoring M1 polarization, as recently reviewed [194]. One of the major anti-inflammatory effects in the inhibition of M1 function consists in the MT_1_ receptor-mediated activation of JAK2 (Janus kinase 2), which phosphorylates STAT3 (signal transducer and activator of transcription 3) [194,195,196,197]. In the nucleus, pSTAT3 dimers activate SOCS1 (suppressor of cytokine signaling 1), which favors NF-κB degradation by virtue of its property as an E3 ubiquitin ligase and, thus, inhibits NF-κB actions at the chromatin [194,198,199,200]. Additionally, suppression of NF-κB actions has been reported for RORα [194,201]. This relationship is highly convincing, since RORα knockout causes strong upregulations of proinflammatory cytokines [202]. However, as RORα has been definitely shown to be incapable of binding melatonin [203,204], actions of melatonin via this transcription factor have to be of indirect nature [18,194]. A possibility of particular interest concerns an effect of SIRT1 on RORα, in its function as a partial mediator of melatonin effects. As far as melatonin upregulates SIRT1, a known mechanism can become effective, which comprises deacetylation of PGC-1α (peroxisome proliferator-activated receptor-γ coactivator-1α) and facilitates the binding of RORα to its response elements (ROREs) [41]. With regard to the number of documented anti-inflammatory actions of SIRT1 summarized above, this idea is insofar attractive as SIRT1 was shown to suppress NF-κB signaling, as likewise reported for RORα [153,154,155]. This does not exclude additional actions of SIRT1, which was also found to be involved in SOCS1 signaling [205]. 

M2 polarization of macrophages, and presumably in similar ways of microglial cells, is largely promoted by STAT6 phosphorylation, in pathways regulated by melatonin and anti-inflammatory cytokines, especially IL-4 and IL-13 [194]. A key step in these processes is tyrosine phosphorylation of IRS-2 (insulin receptor substrate 2), which can be achieved in multiple ways [194]: (1) via IL-4 or IL-13 binding to IL-4 receptor-α, phosphorylation of JAK1/3 or JAK1/Tyk2 (tyrosine kinase-2), which may cause tyrosine phosphorylation of STAT6 directly or, alternately, IRS-2, followed by GRB2 (growth factor receptor bound-2) activation and STAT6 phosphorylation, or (2) via melatonin and an MT_1/2_-dependent cascade of pIRS-2, GRB2, and pSTAT6. pSTAT6 dimers are responsible for M2-specific gene expressions, partially in conjunction with KLF4 (krüppel-like factor 4), which interacts with pSTAT6. Additional effects of melatonin seem to contribute, such as MT_1/2_-dependent inhibition of PI3K (phosphoinositide 3-kinase), thereby blocking the proinflammatory Akt/mTORC1 cascade [194]. Moreover, this inhibitory effect of melatonin can be assumed to prevent two other negative modulatory actions, (1) by the mTORC1 downstream factor GRB10 and (2) by p70S6K (p50S-6-kinase). Tyrosine phosphorylation of IRS-2 and expression of M2 genes were found to be substantially increased by GBR10 knockdown [206]. Serine phosphorylation of IRS-2 by p70S6K was shown to counteract M2 polarization [206].

## 4. Modulation of the Networks by Noncoding RNAs, an Emerging Field

The discovery of countless noncoding RNAs (ncRNAs) with regulatory properties has substantially changed our understanding of regulation. Many of these RNAs interfere with posttranscriptional processes. This is especially the case in most microRNAs (miRNAs), which target mRNAs, and snoRNAs (small nucleolar RNAs), which are involved in RNA processing [207,208]. Other categories of RNAs such as eRNAs (enhancer RNAs) or super-enhancer lncRNAs (super enhancer long noncoding RNAs) directly interact with the chromatin and even with DNA [209]. piRNAs (PIWI-interacting RNAs) silence transposable elements via both transcriptional and posttranscriptional mechanisms [210]. Multiple functions are known for lncRNAs, in addition to enhancer properties. They may either positively or negatively regulate gene expression, which has been even observed in their subgroup of asRNAs (antisense RNAs). Moreover, various lncRNAs are precursors of miRNAs or snoRNAs [209]. Moreover, many lncRNAs and circRNAs (circular RNAs) serve as miRNA sponges and gain particular relevance in the transmission of intercellular signals by exosomes and ectosomes [209,211,212]. With regard to melatonin, numerous ncRNAs of different categories were shown to be influenced by this pleiotropic regulator [209,213,214,215]. As ncRNAs also modulate inflammation, macrophage activities, and NF-κB signaling [216,217,218,219], the relationship to melatonin is of particular interest and will certainly gain increasing future importance. 

A complete consideration of all ncRNAs with positive or negative actions on inflammation would go beyond the scope of this article. A comprehensive list of miRNAs that affect pro- or anti-inflammatory cytokines has been recently published [220]. With regard to the melatonin-related task of this review, only those ncRNAs shall be discussed that are influenced by melatonin or change its downstream factors or interfere with major regulatory parts of the networks, especially if they concern areas of action known to be a matter of beneficial actions by melatonin, such as sepsis, ischemia/reperfusion, aging, or neurodegeneration. Findings obtained in the field of microRNAs are summarized in Table 1. Only those miRNAs have been considered that are related to the field of inflammation regulation. Many additional studies have revealed effects of melatonin on other miRNAs [214,221,222]. However, the outcome in a non-inflammatory context cannot be expected to be the same. This concerns especially the situation in cancer, although cancer can also have an inflammatory aspect. The major difference between non-tumor and tumor cells concerns the inverse relationship between melatonin and SIRT1 and the pro-apoptotic activity of melatonin in tumor cells [5,16,17]. One of the most extensive studies on melatonin effects on miRNAs [221] that has been omitted from Table 1 has been conducted in breast cancer cell lines. The determinations revealed 12 miRNAs that were upregulated by melatonin and 10 others that were downregulated. The analysis of their 5′-utr sequences indicated that these 22 miRNAs might target 2029 mRNAs. 

The pleiotropic targeting of various mRNAs by a single miRNA does not only open the possibility of jointly downregulating several mRNAs that are functionally connected, but may also reflect multiple functions in different contexts. This problem of overlapping actions of miRNAs with roles in both inflammation and cancer is also evident in the findings summarized in Table 1. Although the miRNAs were selected because of their modulation of inflammation, several of them exhibited other, perhaps independent, actions related to tumor promotion and progression. In fact, several molecules listed have been classified as oncomiRs, such as miR-21, miR-23a, miR-29, miR-106b, miR-125b, miR-155, and miR-182 [316,362,363,364]. 

The examples in Table 1, in which changes of miRNA expression by melatonin have been combined with actions of inflammation-related miRNAs on expression of SIRT1, Nrf2, and NF-κB, do not show a uniform picture. One of the reasons concerns the differences in context, cell types, and conditions, under which the studies have been performed. In many cases, changes by melatonin and effects on SIRT1, Nrf2, and NF-κB have been investigated in different systems. In a few cases, opposite effects have been obtained. In addition to organ specificity of melatonin actions, the difference between pro- and anti-inflammatory actions of melatonin has to be considered. The main purpose of this table is to provide information to investigators on which miRNAs they may focus on when studying melatonin effects in inflammation, especially in those cases where actions of melatonin on miRNAs have not yet been analyzed. 

A few special types of connections between melatonin and the three possible downstream factors shall be briefly discussed. The levels of miR-7 have been shown to increase during aging, and it was assumed that it may downregulate SIRT1 [223]. On the other hand, the upregulation of Nrf2 and downregulation of NF-κB in the non-aging context of training athletes, which jointly indicate an antioxidant and anti-inflammatory action, is not easily compatible with reduced SIRT1 in senescence. In other cases of reduced SIRT1 expression, the reservation has to be made that decreased SIRT1 expression may be associated with elevated SIRT1 activity [23], as long as NAD^+^ levels or inhibition by sirtuin inhibitors have not been determined. In reports in which upregulation by melatonin was associated with downregulation of SIRT1 (miR-30a) or Nrf2 and upregulation of NF-κB (miR-146a), this combination may be interpreted as a route of melatonin’s proinflammatory arm. The example of miR-24, which is suppressed by melatonin, but enhances SIRT1 expression, may be taken as a tumor-specific relationship, because this would conform to melatonin’s known suppression of SIRT1 in cancer cells [5,365]. Therefore, this may not be applicable to inflammation control. The presence of miR-126, which is upregulated by melatonin and increases SIRT1 expression, indicates a role of this sirtuin as a mediator of melatonin and may appear, at first glance, as an example of the anti-inflammatory route. However, this contrasts strongly with the upregulation of NF-κB and would require clarification by studying all these factors in the same system. A number of SIRT1-targeting miRNAs is downregulated by melatonin, such as miR-23a, miR-34a, miR-152, miR-155, miR-212, and, according to some but not all data, miR-200a. This reduction of SIRT1-suppressing miRNAs would, again, be compatible with findings on SIRT1-mediated melatonin effects [17,18]. Unfortunately, the findings concerning miRNA-mediated changes of NF-κB are, to date, often not generally compatible with the alterations in the other factors. Further studies will be required for obtaining a coherent picture. However, this reservation concerns only the participation of miRNAs, whereas the effects of melatonin and SIRT1 on Nrf2 and NF-κB have been unequivocally demonstrated [17]. 

Available information concerning melatonin effects on other noncoding RNAs is, unfortunately, limited. A few publications have addressed such actions on lncRNAs. In the case of the lncRNA *H19*, a relationship to miR-675 (cf. Table 1) exists, which is a derivative of the former. The effects of melatonin on H_2_O_2_-exposed cardiac progenitor cells correspond to those of the microRNA and, consequently, *H19* knockdown abolishes the action of miR-675 [355]. The molecule miR-675 was shown to target *USP10* (ubiquitin carboxyl-terminal hydrolase 10) mRNA, which was concluded to cause downregulation of p53 and p21, thereby inhibiting premature senescence by oxidative stress [355]. Upregulation of *H19* and miR-675 by melatonin was also observed in protection experiments against brain injury after subarachnoid hemorrhage [357]. In a study on pulmonary hypertension, melatonin reduced *H19* expression and, therefore, also that of miR-675-3p, whereas *H19* was shown to suppress miR-200a [322]. Meanwhile, numerous other studies have confirmed the role of *H19* in inflammatory processes (not cited), however, they did not consider melatonin. Another type of interaction between lncRNA and miRNA was based on sponging. The lncRNA *MEG3* was reported to enhance pyroptosis in atherosclerotic models, such as human aortic endothelial cells exposed to oxidized LDL (low density lipoprotein). *MEG3* was found to sponge miR-223 and to activate NLRP3, whereas melatonin was shown to reduce pyroptosis by suppressing NLRP3 activation and downstream factors, such as NF-κB, gasdermin D, IL-1β, and IL-18 [366]. In hair follicle fibroblasts of cashmere goats, melatonin upregulated the lncRNA *MTC*, which was associated with proliferation and also NF-κB activation [367]. This finding was in contrast to many other results on NF-κB suppression by melatonin obtained in the context of antioxidant actions. Two other reports have dealt with melatonin’s actions in hepatocellular carcinomas. In one study, melatonin was shown to reduce cancer progression by upregulating FoxA2, which induced the lncRNA *CPS1-IT1*, with a downstream effect of reduced HIF-1α activity [368]. In another investigation, melatonin suppressed DNA repair capacity by upregulating the antisense lncRNA *RAD51-AS1*, which interacts with *RAD51* mRNA, and the findings were interpreted as a sensitization to chemotherapeutic drugs [369]. A functionally different lncRNA, *TERRA* (telomeric repeat-containing RNA), has been investigated in the context of melatonin. This RNA interacts with PARP-1 (polyADP-ribose polymerase-1), a sensor of DNA damage. This interaction was shown to stimulate SASP, but melatonin suppressed SASP-related gene expression and, therefore, an important aging-promoting process [122]. 

Work on the mediation of melatonin effects on lncRNAs can be expected to become highly important in the future [198,370], although this line of investigation is still in its infancy. This assumption is also based on the increasing body of evidence in related fields, in particular, the circadian system [209,371] and gerontology [372,373]. Numerous lncRNAs, e.g., over 600 in murine liver, were found to vary in a circadian fashion, often at very high amplitudes [371]. It would be a surprise if the chronobiotic regulator melatonin would not modulate at least a fraction of them. Among the lncRNAs, particular attention should be paid to subforms of a different functionality, such as asRNAs, eRNAs and, importantly, super-enhancer lncRNAs, all of which contain numerous species under circadian control [209,371]. The prominent role of lncRNAs in the control of inflammation has also become evident and has been reviewed multiple times [374,375,376,377,378,379,380]. In this context, more research on melatonin is desired. A further category of RNAs that will gain importance in the melatonin field, but are still devoid of pertinent investigations, are the circRNAs, which are known to sponge miRNAs [211,381,382,383]. With regard to a miRNA discussed above, miR-7, which is sponged by the circRNA *CDR1as* (alias *ciRS-7*), a few recent publications indicate relationships to NF-κB signaling [384,385,386] and to Aβ secretion [384], and they also demonstrate the profoundness of *CDR1as* deficiency in the brain [387].

## 5. Conclusions

The decrease of melatonin in aging and in various aging-related diseases [1,2] has different, potentially opposite, consequences. The same is valid for melatonin replacement therapies. This results from the dual roles of melatonin as both a pro- and anti-inflammatory regulator [15,16,17]. The conditionality that determines whether melatonin promotes the one or the other mode of immunological response should be a precondition for appropriately judging what kind of change has to be expected. Unfortunately, to date, this conditionality is not completely understood. Tendentially, one can conclude that anti-inflammatory actions of melatonin are mostly prevailing [17], however, with the important exception of autoimmune diseases, in which the immune stimulatory properties of melatonin may turn out to be detrimental. This concerns especially rheumatoid arthritis [50,51,52] and multiple sclerosis [53]. Whether melatonin may be unfavorable in type 2 diabetes of humans, in spite of its clearly antidiabetic actions in rodents, remains to be clarified in detail and may partially depend on age [5]. The difference between humans and rodents has to be seen in the contrasting associations of melatonin with food consumption and related metabolic activities in diurnality vs. nocturnality [5,16]. The inflammatory aspect of type 2 diabetes extends to brain insulin resistance, which has been identified as a proinflammatory change in early AD [107,108,109]. Whether or not melatonin may be detrimental in PD has been a matter of controversy [388,389,390]. The reported improvements by melatonergic antagonists in PD [390] should at least be regarded as a caveat [391].

Under other conditions, melatonin has been shown to preferentially exert anti-inflammatory, antioxidant, and other beneficial actions in aging [10,26,70,74,93,94,95,96,97,98,392,393,394]. Concerning anti-inflammatory effects, melatonin suppresses various processes that lead to enhanced formation of reactive oxygen and nitrogen species and to proinflammatory signaling, as summarized in Section 2. Moreover, it also stimulates several anti-inflammatory pathways and cellular changes that favor this side of the immune system and promote healing, as outlined in Section 3. Among these activities, the promotion of macrophage polarization towards the M2 type may be of particular importance for the shift from pro- to anti-inflammatory behavior, an aspect that has recently received increased attention [137,194,197]. Another potentially decisive action of melatonin concerns the upregulation of SIRT1 in nontumor cells, especially observed in aging animals [3,5,17,18,28,70,395]. Apart from being a relevant factor in aging, SIRT1 displays antioxidant and anti-inflammatory properties (Section 3). As mentioned above, various actions of melatonin are abolished by sirtuin inhibitors or *Sirt1* siRNA. Moreover, some mitochondrial actions of melatonin were shown to be absent in Sirt1^−/−^ mice [396]. The inclusion of SIRT1 and, perhaps, other sirtuins into the spectrum of melatonin’s actions represents an important step forward in the understanding of its aging- and inflammation-related properties. However, one cannot expect that all SIRT1 actions mediate melatonergic regulation. SIRT1 is also controlled by various other factors, including accessory components of circadian oscillators that regulate NAMPT expression and NAD^+^ levels, hormones such as triiodothyronine and glucocorticoids, oncogenes, lncRNAs such as *HOTAIR*, and various miRNAs not regulated by melatonin. The incoherence of melatonin effects on miRNAs and their targeting of typically otherwise melatonin-controlled transcription factors, such as Nrf2 and NF-κB, is evident from Table 1. Moreover, several miRNAs up- or downregulated by melatonin do not cause a rise in SIRT1. However, in the latter case, one has to remain aware that melatonin downregulates SIRT1 in cancer cells, and that several of the miRNAs have been studied in tumors. The selection of miRNAs in Table 1 was restricted to those with demonstrated functions in the control of inflammation. More extensive studies on melatonin, SIRT1, and miRNAs will presumably reveal additional cases that are related to the immune system and also to aging and age-related diseases. As examples, the associated roles of SIRT1 and miRNAs in SASP and in Aβ toxicity shall be briefly mentioned [125,397]. MicroRNAs and other noncoding RNAs have brought about a new, considerably expanded level of complexity into the relationships between melatonin, inflammation, and aging. There is considerable difficulty that arises from multiple mRNAs targeted by a single miRNA species. A further level of complexity in the regulatory networks can be expected as soon as the intercellular communication, via exosomal RNAs, is more profoundly understood in its details and its variations according to diseases and aging.

## Figures and Tables

**Table 1:** Regulation of microRNA expression by melatonin and effects of microRNAs on SIRT1, Nrf2, and NF-κB in the context of inflammation.

miRNA	Change by Melatonin	Effect on Sirt1	Effect on Nrf2	Nrf2-Related Target	Effect on NF-κB	NF-κB-Related Target
miR-7		↓? [223]	↑ [224]	Keap1	↓ [225,226]	
miR-7-5p					↓ [227]	
miR-9		↓ [228,229,230,231,232,233,234,235,236]			↓ [236,237,238,239,240,241,242]	
miR-9					↑ [243,244]	MCPIP1, TRIM56
miR-20a					↑ [245,246]	CYLD
miR-21		↓ [247]				
miR-23a	↓ [194,248]	↓ [249,250,251]	↑ [252]	Keap1	↑ [194]	
miR-23a-3p		↓ [253]				
miR-23b		↓ [249]				
miR-23b-3p		↓ [155,254]				
miR-24	↓ [255]	↑ [256]				
miR-24-3p			↑ [257]	Keap1		
miR-26a					↓ [258]	
miR-27b					↓ [259]	
miR-29		↓ [260]	↑ [261,262]	Keap1		
miR-30a	↑ [263]	↓ [264]			↓ [265]	
miR-30a-3p		↓ [266,267]				
miR-30e-5p	↓ [268]					
miR-31					↓ [269]	TRADD
miR-34a	↓ [270]	↓ [271,272,273,274,275]				
miR-34a-5p		↓ [276]				
miR-101			↓ [277]			
miR-106a/b		↓ [278]				
miR-124a		↓ [279]				
miR-125a/b					↑ [280,281,282,283,284]	A20
miR-125b					↓ [285,286,287,288,289]	TRAF, MIP-1α
miR-126	↑ [270]	↑ [290]			↑ [291,292,293]	TOM1, IκB
miR-128		↓ [294,295,296]			↑ [297,298]	IκB
miR-132		↓ [249]				
miR-135a		↓ [228]				
miR-142-3p	↑ [299]					
miR-144			↓ [300]			
miR-145-5p		↓ [276]				
miR-146a	↑ [270]		↓ [301]		↑ [302,303,304,305]	TRAF6, IRAK1
miR-150					↓ [306,307,308,309]	
miR-152	↓ [310]	↓ [256]				
miR-153			↓ [300]			
miR-155	↓ [194,310]	↓ [279,311,312]	↑ [313,314]			
miR-181a-c		↓ [228,230,315]				
miR-182		↓ [316]			↑ [317,318,319,320]	CYLD, TCEAL7
miR-195-5p		↓ [276]				
miR-199b		↓ [228]				
miR-200a	↑ [321,322]	↓ [189,323,324,325,326,327]	↑ [328,329,330,331,332,333,334,335,336]	Keap1	↑ [337]	
miR-200a-3p		↓ [276,338,339]				
miR-204		↓ [228]				
miR-210					↓ [340,341]	DR6
miR-212	↓ [299]	↓ [249,267,342,343,344]				
miR-217		↓ [345]				
miR-301a					↑ [346,347,348,349,350]	NKRF
miR-326					↑ [351]	BCL2A1
miR-340			↓ [352]			
miR-340-5p			↓ [155,300,353]			
miR-495		↓ [354]				
miR-675	↑ [355]					
miR-675-3p	↑ [322]					
Let-7					↑ [356]	A20
Let-7a	↑ [357]					
Let-7e					↑ [358]	IκBβ
Let-7f					↑ [359]	A20
Let-7g					↑ [360]	
Let-7i		↓ [361]			↑ [361]	SIRT1

Abbreviations: A20, ubiquitin-editing enzyme A20 (=TNFAIP3, tumor necrosis factor alpha-induced protein 3); BCL2A1, B-cell lymphoma 2A1; CYLD, cylindromatosis; DR6, death receptor 6; IRAK1, interleukin-1 receptor-associated kinase 1; KEAP1, Kelch-like ECH-associated protein 1; MCPIP1, monocyte chemotactic protein-induced protein 1; MIP-1α, macrophage inflammatory protein-1α ; NKRF, NF-κB repressing factor; TCEAL7, Transcription elongation factor A protein-like 7; TOM1, target of Myb1; TRADD, tumor necrosis factor receptor type 1-associated DEATH domain protein; TRAF6, tumor necrosis factor receptor-associated factor 6; TRIM56, tripartite-motif-containing protein 56. ↓↑ indicate down- or upregulations, respectively.
